# Engineering and Exploiting Immobilized Peptide Organocatalysts for Modern Synthesis

**DOI:** 10.3390/molecules30122517

**Published:** 2025-06-09

**Authors:** Marco Francescato, Hang Liao, Luca Gentilucci

**Affiliations:** 1 Department of Chemistry “G. Ciamician”, University of Bologna, Plesso Navile—Ue4, via Gobetti 85, 40129 Bologna, Italy; marco.francescato2@unibo.it (M.F.); hang.liao2@unibo.it (H.L.); 2 Health Sciences & Technologies (HST) CIRI, University of Bologna, Via Tolara di Sopra 41/E, 40064 Ozzano Emilia Bologna, Italy; 3 Center for Chemical Catalysis—C3, University of Bologna, via Gobetti 85, 40129 Bologna, Italy

**Keywords:** peptide organocatalyst, green chemistry, β-turn, silica, polystyrene, PEG, absorption, asymmetric catalysis

## Abstract

Short- and medium-sized peptides have long been used as effective and versatile organocatalysts. In the early 80s, Inoue used diketopiperazines in the Strecker reaction, while Juliá and Colonna reported the epoxidation of chalcone catalyzed by poly-L-Ala. Since then, a variety of peptide-catalyzed reactions have been described. However, peptide synthesis typically implicates the use of toxic reagents and generates wastes; therefore, peptide recycling is expected to significantly improve the overall sustainability of the process. Easy recovery and recycling of peptide catalysts can be expediently attained by covalent binding, inclusion, or adsorption. In addition, immobilization can significantly accelerate the screening of new peptide catalysts. For these reasons, diverse supports have been tested, including natural or synthetic polymers, porous polymeric networks, inorganic porous materials, organic-inorganic hybrid materials, and finally metal–organic frame-works.

## 1. Introduction

In the field of biologically active compounds, oligopeptides are currently regarded as a good compromise between proteins and small molecules. Short peptide sequences composed of just 3 or 4 residues show the most favorable ratio between bioactivity and size [1]. Similarly, oligopeptides can mimic the minimal functional units of enzymes, enabling regio- and stereoselective transformations of substrates. This unique capability makes them particularly attractive as organocatalysts [2]. 

The use of peptides as catalysts dates back to the period around 1980 when Juliá and Colonna utilized poly-L-alanine for the epoxidation of chalcone, and Inoue et al. utilized diketopiperazines in the asymmetric synthesis of cyanohydrins. The Juliá–Colonna (J-C) epoxidation is one of the most thoroughly investigated processes promoted by poly-amino acids [3,4]. Electron-poor olefins, such as chalcones, are oxidized with H_2_O_2_ in a water/organic solvent mixture in the presence of a base and a homo-oligopeptide. Peptides composed of just one amino acid were conveniently synthesized through the polymerization of N-carboxyanhydrides (NCAs). These are cyclic amino acid derivatives in which the carboxy group is activated while the amino group is protected. Due to their high reactivity and tendency to polymerize, NCAs can be employed for the synthesis of hetero-oligopeptides only under strictly controlled reaction conditions [5]. 

**Figure 1:**
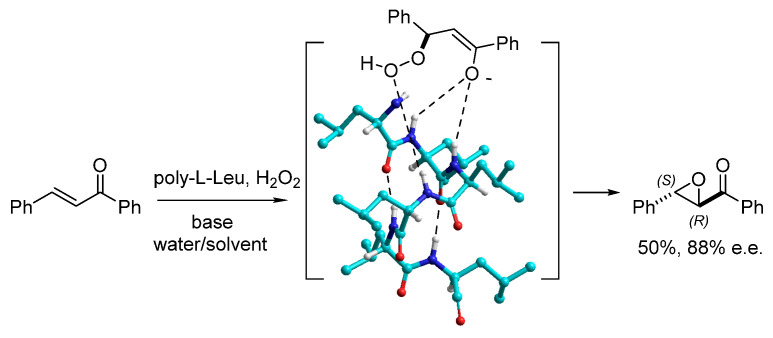
Juliá–Colonna epoxidation of chalcone catalyzed by polyLeu, and a sketch of the proposed substrate-catalyst complex.

The J-C reaction commences with the nucleophilic addition of peroxide to the α,β-unsaturated ketone, followed by the ring closure of the resulting enolate. The reaction mechanism was deeply investigated [6,7]; as it turned out, the diastereoselectivity of both steps (up to 96% e.e., and up to 96% yield) is the result of the interactions between the reactants and the peptide. In the proposed model, as reported in Figure 1 for polyLeu [8], the peptide adopts an α-helical conformation, and the reactants are held in place through hydrogen bonding interactions with the N-terminal amide groups.

**Figure 2:**
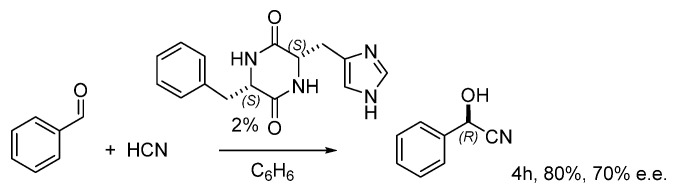
Asymmetric addition of hydrogen cyanide to benzaldehyde catalyzed by DKP.

In the same years, Oku and Inoue reported the peptide-catalyzed asymmetric addition of HCN to benzaldehyde [9], and the best results were obtained with 2 mol% of Phe-His diketopiperazine (DKP). In 30 min, the product could be formed in 40% yield and 90% e.e., but it rapidly racemized over prolonged reaction times until 80% yield and 70% e.e. were obtained after 4 h (Figure 2). The reaction mechanism was investigated by diverse authors, and the most convincing kinetic study supported a second-order process in DKP.

The use of proline for reactions involving enamine intermediates boomed after List et al. reported the proline-catalyzed direct asymmetric aldol reaction [10]. In the following decades, a number of amino acid- [11] or peptide-catalyzed reactions have been proposed [12,13,14]: aldol condensations, conjugate/Michael additions, Mannich reactions, epoxidations, acylation for the desymmetrization of diphenols or diols, desymmetrization by silylation, atroposelective bromination, site-selective polyene oxidation, enantioselective [2+2] photocycloaddition, light-driven deracemization of cyclic ureas, etc.

Miller and co. reviewed the literature on peptide-catalyzed asymmetric reactions in 2007 [15] and reviewed later developments in 2020, with particular emphasis on structural and mechanistic aspects [16].

In addition to the above-discussed catalysts, peptides have also been utilized as scaffolds for the preparation of metal–peptide hybrid catalysts [17]. On the other hand, a variety of self-assembling peptides, including surfactant peptides, amyloid peptides, and lipopeptides, can spontaneously organize in aqueous environments into diverse nanostructures such as fibrils, nanotubes, coiled-coil bundles, and micelles. These supramolecular structures can serve as scaffolds for the precise spatial arrangement of catalytic residues and cofactors, enabling transformations that are otherwise challenging or unconventional [18,19,20].

Peptides carrying one or two prolines at the N-terminus (Pro- and Pro-Pro-peptides) deserve a separate discussion since they represent a significant fraction of the peptide catalysts. Pro and other chiral secondary amines are able to activate the carbonyl group of aldehydes and ketones via either (1) iminium ions for stereocontrolled nucleophilic attack or (2) enamines for stereoselective addition to electrophiles. Wennemers and others proposed that Pro-Pro-Glu (homo or heterochiral) and similar tripeptides can be utilized as catalysts of 1,2- and also 1,4-addition reactions. Indeed, the distance between the N-terminal secondary amine and the carboxylic group is increased by about 3 Å as compared to simple amino acids, and this additional distance consents to forming stable complexes with longer electrophiles [21,22]. In contrast to helix-forming polyAla or polyLeu oligomers (Figure 1), these Pro-Pro-Glu sequences adopt in solution clear β-turn conformations, with the carboxylic acid moiety in the side chain of the Glu residue acting as a “lid that can close to restrict the conformational space of the catalyst”. Yet, the intermediate enamine still adopts predominantly a β-turn but is significantly more flexible [23].

In addition to the literature reported in the paragraphs above, several other relevant reviews and papers about homogeneous peptide organocatalysis have been published. These reviews nicely and exhaustively cover the state of the art of the matter and discuss in-depth performances and mechanisms [24,25,26,27].

Tables reporting the catalytic performances among different immobilized peptide organocatalysts for direct comparison can be found in the following References: [15,16]. Therefore, this review is mainly dedicated to the specific field of immobilized peptide organocatalysis, focusing in particular on the immobilization strategies and their applications.

## 2. Heterogeneous Peptide Organocatalysis

Peptide synthesis, especially solid-phase peptide synthesis (SPPS), is notably lacking in green chemistry principles, largely due to its poor atom economy, reliance on large excesses of reagents, and the use of toxic organic bases, such as triethylamine or piperidine. Additionally, SPPS generates significant chemical waste through non-recyclable resin supports and large volumes of hazardous organic solvents, e.g., dichloromethane dimethylformamide [28,29]. Considering the significant environmental impact of peptide synthesis, it is essential that if a peptide is to be used as an organocatalyst, it can be recycled multiple times. Reusability not only helps offset the environmental burden of the synthesis but also contributes to making the overall process more economically efficient.

In this context, the immobilization of peptide catalysts onto a solid support represents a fundamental strategy. Very often, the new catalysts that are being currently proposed are designed as solid-supported materials. The main advantage is that the reaction product can be separated and isolated by simple filtration or centrifugation, and the peptide catalyst can be recovered and recycled.

While the immobilization of proline derivatives appears to be quite expedient [30,31], success in the immobilization of a peptide can be challenging [32,33]. Among the pitfalls, it must be mentioned that quite often, the supported peptides show a decrease in efficiency as compared to the non-supported peptides. Indeed, the solid support or the linkage can interfere with catalyst functions. Hence, selecting the appropriate linker to connect the catalyst to the solid support, along with the position at which they are bound, is of utmost importance. Another main drawback is the deactivation of the organocatalyst after a few cycles of reuse due to chemical modification or impediment of the catalyst’s active site caused by the reactants, the products, or the byproducts. A third limitation is that many catalytic systems require the use of acidic or basic additives or co-catalysts; hence, filtration is not enough to afford the isolated products. In summary, the design of a properly immobilized organocatalyst requires the choice of the appropriate solid support, the nature and length of the linker, the type of reaction employed for immobilization, and the display and orientation of the catalyst onto a solid support. The advantages and disadvantages of each type of support, either polymeric, inorganic, or hybrid, are discussed in the following sections.

**Figure 3:**
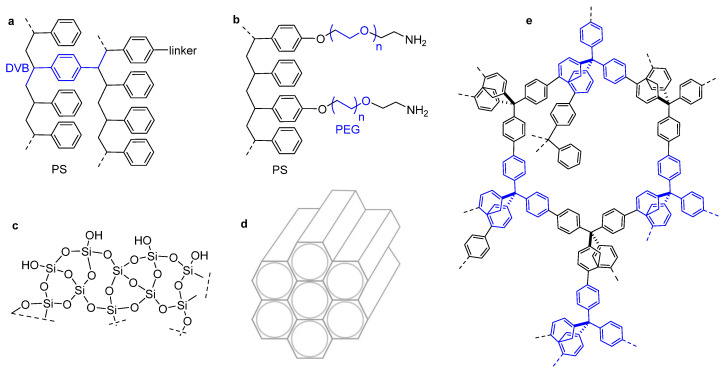
Sketches of the structures of (**a**) polystyrene (PS) cross-linked with divinylbenzene (DVB), (**b**) polyethylene glycol grafted on polystyrene (PEG-PS), e.g., TentaGel S-NH_2_ resin, (**c**) silica microstructure, (**d**) ordered mesoporous silica (OMS), (**e**) an example of microporous polymer network.

Among the preferred materials generally employed as supports, it is possible to mention organic polymers of natural origins, such as chitosan, as well as synthetic organic polymers including cross-linked hydrophobic polystyrene (PS), hydrophilic polyethylene glycol (PEG)-PS copolymers, polyacrylamide, polyethylene glycol-polyacrylamide (PEGA), etc. [34], but also inorganic materials such as mesoporous silica, hydrotalcite, etc. [35] (Figure 3).

The development of fully organic materials with high surface areas—such as microporous polymer networks (MPNs) [36] and covalent organic frameworks (COFs) [37] —greatly expanded the potential of organic nano- and mesoporous systems, opening new avenues for their use in heterogeneous catalysis.

In contrast to almost all other microporous materials, MPNs are highly cross-linked fully, covalent structures exclusively built up from organic matter. COFs are a distinct group of crystalline and porous organic polymers characterized by stable porosity and well-organized architecture (Figure 3). Compared to other porous materials, these classes can be precisely designed and reliably synthesized. Diverse strategies for pre- and post-synthetic modifications have been explored to consent to their application in heterogeneous chiral catalysis.

Recently, scientists have begun to immobilize organocatalysts also on metal–organic frameworks (MOFs). MOFs are low-density, three-dimensional arrays composed of inorganic nodes (metal ions or clusters) linked by polytopic functional organic struts, characterized by high surface area (up to 10,400 m^2^/g) and large pores (98 Å) [38].

The immobilization of the organocatalysts has found rather frequent application in two particular sectors: the ‘one-bead one-compound’ (OBOC) method and continuous flow chemistry. The OBOC method is particularly useful for rapid screening of peptide catalysts. The libraries are synthesized on beads by a combinatorial ‘split synthesis’ method. After each step, a new residue is added until each bead displays only one peptide sequence. This bead-supported library is then assayed for a specific activity. Alternatively, the compound on each bead is released from the solid support via a cleavable linker for subsequent solution-phase tests. The active peptides are then processed by an automatic protein sequencer for sequence determination or analyzed by mass spectrometry [39].

In continuous flow chemistry, a solution of the reagents is forced to flow through a bed of immobilized organocatalysts. The heterogeneous organocatalyst is usually placed into a packed columnar reactor in the form of a swellable microporous peptide resin, as a block of macroporous material containing the peptide, or even as a 3D printed material.

Alternatively, the peptide is covalently attached to the reactor’s internal walls. This method has important advantages over traditional batch procedures [40,41]. First, the product is not contaminated with the catalyst. Second, if the catalyst proves robust enough, the effective catalyst loading becomes a function of operation time, leading to extremely high turnover numbers (TONs) for the overall process even when small-size devices are operated for long periods of time, whereas at any given time the substrates are exposed to an excess of catalyst inside the reactor and this allows high conversions in short contact times.

As an alternative to peptide immobilization, the peptide catalyst itself can be heterogenized for easy recycling by simple filtration after the reaction. For instance, high yield and enantioselectivity were obtained in J-C epoxidation of chalcone using an easily recoverable polyLeu peptide equipped with an N-terminal imidazolium (96%, 95% e.e.) [42]. This polyLeu-imidazolium was formed by polymerization of NCAs promoted by an aminomethyl initiator incorporating the imidazolium. Compared to classical J-C catalysts, this insoluble, powdery catalyst reduced the reaction time, and the recycled catalyst could be reused for seven cycles without deterioration in catalytic efficiency.

Besides the bibliography as presented in this Section, other relevant reviews and papers already appeared on heterogeneous catalysis by peptides can be found here: [43,44,45].

## 3. Immobilization of Peptide on Polystyrene Resin

Polystyrene cross-linked with divinylbenzene (typically 1%) is the most common core resin utilized in solid-phase peptide synthesis (Figure 3). A variety of functional groups can be included in the polymeric matrix for linking the peptidic sequences. Typically, this hydrophobic material is utilized in the form of beads in aprotic organic solvents, albeit several examples of successful reactions have also been described in aqueous media [46]. Due to noteworthy mechanic resistance, higher loading capacity, and stability to pressure, this resin is often preferred to other copolymer resins for reactions conducted in flow chemistry.

Shortly after Juliá and Colonna proposed the stereoselective peptide-catalyzed epoxidation, the reaction was improved under several aspects. Although a wide range of amino acids has been tested, polyLeu became the standard. Banfi et al. studied the effects of C- and N-terminal modifications of the polyLeu sequence. Among the modifications, the peptide was conjugated to a PS solid support without compromising the efficacy (yield up to 95% and up to 96% e.e.) [47]. Poly-amino acids supported onto poly(styrene-co-divinylbenzene) were also prepared by Itsuno et al. [48] by polymerization of NCAs using a PS resin incorporating aminomethyl functionality as an initiator. These poly-amino acids were tested as chiral catalysts in the epoxidation of benzalacetophenone in toluene/water, and polyLeu gave the best result in this system to obtain 92% yield with 99% ee. Hence, the supported peptide catalyst performed better as compared to the polypeptide alone (see, for instance, Figure 1).

**Figure 4:**
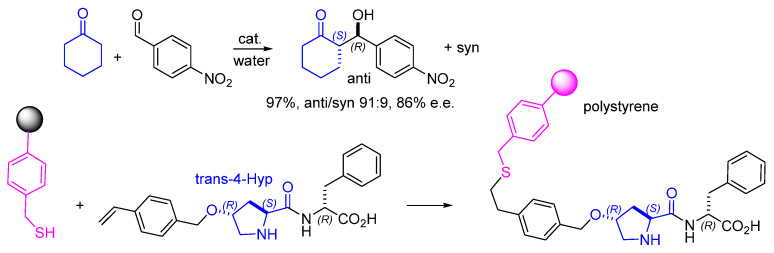
Direct aldol reaction using a supported dipeptide. The dipeptide containing a trans-4-styrenyl-Hyp was immobilized by thiol-ene coupling with mercaptomethyl-functionalized PS resin.

Gruttadauria and co. immobilized proline dipeptides or a Pro-amide derivative by thiol–ene coupling between a mercaptomethyl-functionalized polystyrene resin and a dipeptide containing a trans-hydroxyproline (Hyp) functionalized with styrene at position 4 [49]. These materials were used for the direct asymmetric aldol reaction in water (Figure 4). After the evaluation of various dipeptides and stereoisomers, the supported catalyst bearing a Hyp-D-Phe sequence gave the highest yield (97%), diastereoselectivity (anti/syn 91:9), and enantioselectivity (for anti (*S*,*R*), 86% e.e.) in the aldol reaction between cyclohexanone and 4-nitrobenzaldehyde under aqueous conditions. The catalyst could be reused nine times with reproducible results and activity or could be easily regenerated when its activity was diminished.

**Figure 5:**
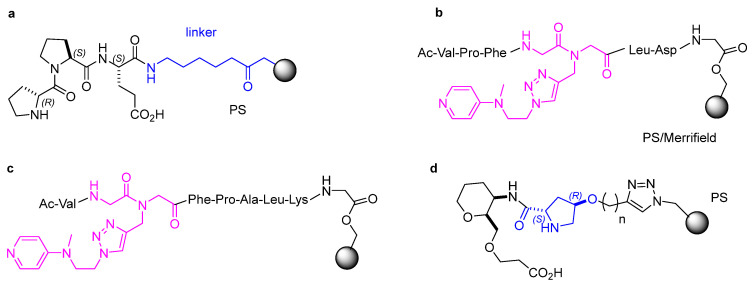
(**a**) Wennemers’ tripeptide catalysts for conjugate additions supported onto PS resins; (**b**,**c**) DMAP–peptide-resin conjugates for site-selective acylation of poly-hydroxylated compounds (the artificial DMAP-amino acid **1** is colored in pink); (**d**) hybrid tetrahydropyrane catalyst supported on PS.

As anticipated in the Introduction, Wennemers and others proposed the use of D-Pro-Pro-Glu tripeptides as catalysts of not only 1,2- but also 1,4-addition reactions [50]. Accordingly, immobilized peptide catalysts containing the D-Pro-Pro-Glu Wennemers’ tripeptide (see also next sections) were successfully employed for the conjugate addition reactions between aldehydes and β-nitrostyrene in flow reaction setups (syn/anti up to >99:1, and up to 98% e.e.) (Figure 5a). Under flow conditions, cross-linked polystyrene (PS) resin was envisioned to be more suitable than TentaGel (TG) because it has a higher loading capacity and swells less than TG, and the system achieved more than 600 turnovers [51].

DMAP–peptide conjugates containing the artificial amino acid 1 (Figure 5b) were supported onto a Merrifield resin. The DMAP moiety was anchored to the peptide by Cu-catalyzed alkyne-azide cycloaddition (CuAAC) reaction. The authors utilized these DMAP–peptides for the site-selective acylation of poly-hydroxylated substrates. The benzoylation of glucose-derived diols, i.e., methyl a-D-glucopyranoside protected at hydroxy groups 5 and 6 via acetal was performed in the presence of diverse DMAP–peptide sequences and Ac-Val-Pro-Phe-**1**-Leu-Asp-(Gly linker)-resin (10 mol%) (Figure 5b) provided the monobenzoylated product at the position 2 in an excellent 96% isolate yield.

On the other hand, Ac-Val-**1**-Phe-Pro-Ala-Leu-Lys-(Gly linker)-resin (Figure 5c) was found to be optimal for the benzoylation of ouabagenin, a steroid derivative with free OH groups at the positions 3,5,11,14. The catalyst exclusively promoted the benzoylation at position 11, with a conversion of 35%. The catalyst was recovered and reused and maintained both activity and selectivity during 11 cycles [52].

Szőllősi and co. utilized di- and tripeptides bound to polystyrene resin through a methyl benzhydryl amine (MBHA) linker in asymmetric aldol reactions [53] between 2-nitrobenzaldehyde or 2-methylpropanal and acetone or cyclohexanone. The reactions represented an interesting case of enantiodivergence when using di- versus tripeptides as catalysts. In all the reactions examined, the solid-supported Pro-Glu dipeptide afforded the (*R*)-configured aldol product (95%, 52% e.e.), while Pro-Pro-Glu tripeptide favored the (*S*)-aldol product (50%, 39% e.e.). The authors proposed that the MBHA linker and resin may have exerted some influence over the reactivity and selectivity of the aldol reaction.

Similarly, Machuca at al. prepared various dipeptidic organocatalysts bound to MBHA resin and evaluated their efficiency in the asymmetric aldol reactions between cyclohexanone and diverse aldehydes. The best results were obtained with the supported Pro-Gly dipeptide (99% conversion, anti/syn 91:9, 66% e.e.), and the catalyst was reused in five consecutive cycles in the asymmetric aldol reaction, albeit with slightly reduced yield and stereoselectivity [54].

García-Monzón et al. proposed the immobilization of hybrid catalysts composed of a dipeptide and a tetrahydropyrane ring onto polystyrene cross-linked with divinylbenzene, using CuAAC reaction (Figure 5d). The catalysts have been used for the enantioselective Michael addition of aldehydes to β-nitrostyrenes, with yields up to 99% and up to 97% e.e. Due to the bifunctional character of these catalysts, the use of additives for regenerating the catalytic activity was not necessary [55]. For instance, for the reaction between nitrostyrene and butanal, the catalyst was recycled four times, and the performance decreased only slightly (from 98% to 89% yield, from 96% to 95% e.e.), while the syn/anti ratio was improved (from 11:1 to 23:1).

**Figure 6:**
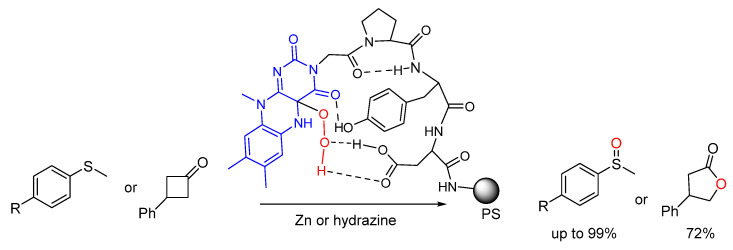
Proposed model for the oxidation catalyzed by flavin-peptide hybrid supported on resin.

Imada and co. proposed FAD-like reactivity with isoalloxazine-peptide hybrids as catalysts supported on PS-amine resin in the oxidation of phenylthioethers or 3-phenylcyclobutan-1-one to the corresponding methylsulfinylbenzene or lactone, respectively, by oxygen. The peptide sequences included Pro at the i+1 position to favor a γ-turn structure and an acidic functionality at the i+3 position for further intra- and intermolecular interactions, hence orienting the peptide scaffold near the catalytically active moiety (Figure 6). The Flavin-peptide hybrids transported the peroxy species generated from O_2_ [56]. The catalyst was restored by reduction with zinc or hydrazine. Interestingly, this system showed great chemoselectivity in the Baeyer-Villiger oxidation of cyclobutan-1-ones (Figure 6). This chemoselectivity was attributed to hydrogen–bonding interaction between the i+3 carboxylic group and the peroxy species to enhance its nucleophilicity towards the cyclobutanones.

Very recently, a tetrapeptide bearing π-methyl histidine (Pmh) as the catalytically active moiety was synthesized by solid-phase peptide synthesis (SPPS) on Wang-resin. The supported peptide was utilized for the site-selective acetylation of different monosaccharides for multiple reaction cycles and was easily reused after separation from the reaction solution. For instance, the catalyst promoted the reaction of 4,6-O-benzylidene-α-D-glucopyranoside with acetic anhydride, giving a conversion of 94% and moderate regioselectivity, 70% in favor of the 2-acyl derivative. The catalyst was used in flow chemistry without loss of reactivity and selectivity [57].

## 4. Immobilization on Polyethylene Glycol-Polystyrene Resins

Polyethylene glycol grafted on polystyrene (PEG-PS), such as the TentaGel^®^ resins, are grafted copolymers consisting of a low cross-linked polystyrene matrix on which polyethylene glycol (PEG) is grafted via an ethyl ether group which increases stability towards acid treatment and minimizes PEG-leaching. The graft copolymer shows modified physicochemical properties with respect to the separated polymers since they have hydrophobic and hydrophilic portions. For these reasons, PEG-PS copolymers are frequently utilized in organocatalysis under aqueous conditions. The main advantage of the use of TentaGel resin is to eliminate complications due to the aggregation of free hydrophobic peptides in solution. These copolymers are reasonably stable to pressure and, therefore, can be used in batch processes as well as under continuous flow conditions.

Aiming at further improving the practicality of the J-C epoxidation of chalcone, PEG-PS-supported 15-mer or 20-mer of Leu were prepared using a peptide synthesizer. These catalysts converted chalcone into the corresponding epoxide in sufficient to good yield and enantioselectivity (up to >97% yield, e.e. up to 89%) [58], and eventually were utilized to prepare the antiamnaesic agent (+)-clausenamide.

Berkessel et al. prepared Leu oligomers of varying chain lengths (from 1 to 20) directly on TentaGel S-NH_2_ resin. Hence, the peptides were linked to the solid support by a robust amide group. The authors demonstrated that five Leu residues were sufficient to catalyze the J-C epoxidation of chalcone with 96–98% e.e. [59].

A PEG-PS resin was also used to support the tripeptide D-Pro-Tyr-Phe, giving a catalyst for the direct asymmetric aldol reaction of acetone with aldehydes in aqueous media in the presence of 20% zinc chloride (conversion up to 100%, and up to 84% e.e.). The peptide catalyst could be separated from the reaction mixture by simple filtration and was reusable at least five times [60].

**Figure 7:**
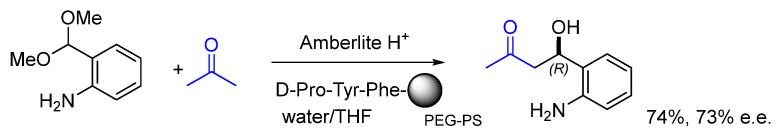
Aldol reactions between acetals and acetone using a one-pot tandem procedure.

Kudo and co. expanded the scope of asymmetric aldol reactions beyond aldehydes by employing acetals as masked electrophiles. These authors developed the first one-pot acid- and base-catalyzed reactions in a single reaction vessel using resin-bound peptide catalysts (Figure 7) [61]. Commercially available Amberlite IR-120 (H^+^-form) was used as an acid catalyst. This resin is a divinylbenzene-crosslinked partially sulfonated gel-type PS. As a base catalyst, a Pro-peptide supported on PEG–PS resin was employed. Using the two resins in tandem, the acetal was hydrolyzed to aldehyde, which in turn underwent the enantioselective aldol reaction with acetone to afford the β-hydroxyketone (74%, 73% e.e. for (*R*)). The catalyst could be easily separated from the reaction mixture by filtration and subsequently regenerated by drying, and the catalytic activity was largely retained after six cycles.

**Figure 8:**
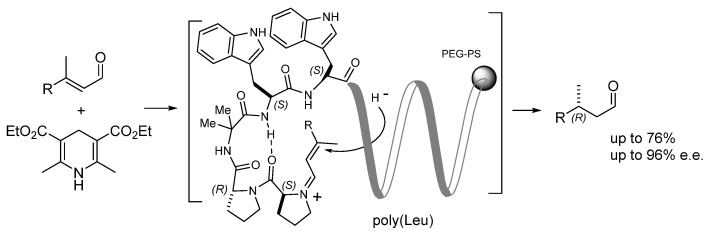
β-Turn-helical peptide catalyst supported on PEG-PS for stereoselective hydrogenation.

In order to provide a hydrophobic environment for attracting organic substrates when reactions were conducted in a mixture of water/THF, the same authors developed a peptide catalyst consisting of the peptide Pro-D-Pro-Aib-Trp-Trp, attached to a polyLeu chain. As discussed in the introduction, Pro-Pro-peptides tend to adopt turn conformations. The polyLeu tether provided a helical hydrophobic cavity in aqueous media that brought about a remarkable acceleration of the reaction (Figure 8). These sequences were supported on PEG-PS resin and utilized in transfer hydrogenation reactions in aqueous conditions. The N-terminal Pro residue worked via iminium catalysis and activated enals in the reduction with Hantzsch ester. As expected, the Pro-peptide itself formed a β-turn and provided the proper chiral environment for asymmetric induction (up to 76% conversion, up to 96% e.e. for (*R*)) [62].

The peptidomimetic strategy proposes different solutions to the design of tri- or tetrapeptides adopting a well-defined β-turn conformation, including the introduction of a D-configured residue at position 2, the use of dehydroamino acids, the use of β-turn mimetic scaffolds (e.g., the Friedinger lactam) and so on [63]. For this reason, oligopeptides adopting this secondary structure motif have been utilized for selective reactions with many different substrate classes.

A similar catalytic system supported onto a PEG-PS resin and composed of the peptide D-Pro-Aib-Trp, as a β-turn element, and a hydrophobic helical polyLeu chain, was shown to bolster asymmetric induction in the α-oxidation of aldehydes by air with TEMPO, in the presence of FeCl_2_ tetrahydrate, and NaNO_2_ (87% yield, 93% e.e.) [64].

**Figure 9:**
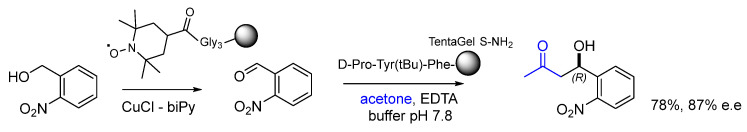
Tandem oxidation/aldol reaction and peptide catalyst supported on TentaGel.

Kudo and co. also used resin-bound peptide-based catalysts to explore a tandem oxidation/direct aldol reaction to be adopted for chemically unstable aldehydes. Indeed, the aldehydes were obtained during the first oxidation step from the corresponding commercially available alcohols (Figure 9) [65]. The tripeptide D-Pro-Tyr(tBu)-Phe was attached to a TentaGel S-NH_2_ resin, while 4-carboxy TEMPO was immobilized on Gly_3_-PEG–PS resin via an amide bond. TEMPO-peptide oxidized the alcohol to the corresponding aldehyde in the presence of Cu(I)-2,2′-bipyridine. Thereafter, the resin-supported D-Pro-Tyr(tBu)-Phe peptide catalyzed the direct aldol reaction between aldehyde and acetone via enamine catalysis to provide the β-hydroxyketone (78% yield and 87% e.e. for (*R*)). The catalyst could be recycled and maintained its activity over eight cycles.

Kudo et al. also revisited the J-C epoxidation, namely the epoxidations of α,β-unsaturated aldehydes or ketones, promoted by hetero-oligopeptides attached to amphiphilic PEG-PS resin, to avoid aggregation or sedimentation caused by hydrophobicity. In the epoxidation of enones by the catalyst Ala-(1-Pyn)-Pro-(Leu-Leu-Aib)_2_-resin, where 1-Pyn = l-3-(1-pyrenyl)alanine, inverted stereoselectivity was observed as compared to the classic J-C reaction (up to 80% yield, up to 88% e.e.) [66]. For the epoxidation of α,β-unsaturated aldehydes, the peptide sequence D-Pro-Ach-[Ala(1-Pyn)]_3_, Ach = aminocyclohexanecarboxylic acid, was connected to a polyLeu helical section, and supported onto resin (up to trans/cis ratio of 98:2, 95% e.e.) [67].

As for the conjugate addition reactions between aldehydes and nitroolefins, Arakawa, Wennemers, and co. immobilized the tripeptide H-D-Pro-Pro-Glu on polystyrene (PS) resin, as well as on hydrophilic TentaGel (polyethylene glycol-PS), and also on polyethylene glycol-polyacrylamide resin (PEGA). The expected efficacy of the homogeneous catalyst was maintained; the best results for the reaction between butanal and β-nitrostyrene were obtained with the TentaGel-supported peptide (quantitative yield, syn/anti ratio >99:1, and 96% e.e.). Finally, the catalyst could be recycled at least 30 times with the same high catalytic performance [68].

**Figure 10:**
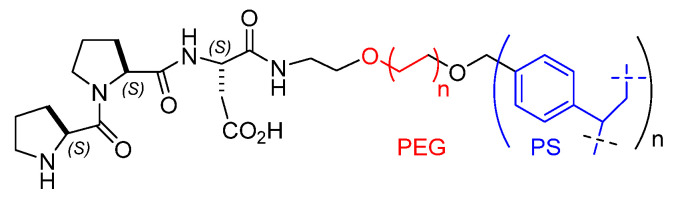
Wennemers’ tripeptidic catalyst for aldol reaction, supported on PEG-PS.

Fülöp and co. developed a highly recyclable, solid-supported peptide for aldol reaction in continuous flow. Inspired by Wennemers’ tripeptidic catalyst, the authors synthesized a Pro-Pro peptide on TentaGel resin via SPPS, which was directly packaged into a cartridge, obviating the need for any purification steps [69]. With the benchmark substrates, this method generated the aldol product in quantitative conversion with 80%, e.e., and the catalyst cartridge was recycled up to 20 times while retaining efficiency and selectivity (Figure 10).

In 2012, Kudo and co. reported a method for the enantioselective silylcyanation of aldehydes employing PEG–PS-bound helical oligoLeu, which afforded TMS-protected cyanohydrins. As a prototypic example, the reaction between benzaldehyde and TMS-CN gave a quantitative yield but moderate enantioselectivity (62% e.e.), while other peptides, i.e., polyAla, polyVal, polyIle, polyGlu(OBzl), gave inferior results. The enantioselectivity of the reaction was highly dependent on the length of the polyleucine chain, indicating that the helicity of the catalyst played a key role [70].

**Figure 11:**
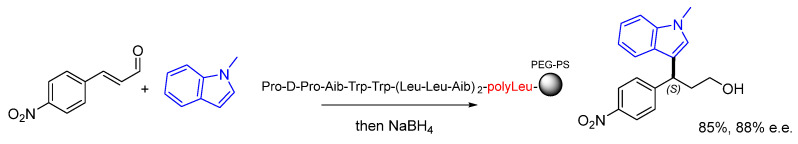
Resin immobilized β-turn-helical peptide catalyst for Friedel–Crafts alkylation of indoles.

In prosecuting their studies, Kudo and co. reconsidered the use of β-turn-forming Pro-D-Pro-peptides, plus a polyLeu α-helical region, in conjugate addition reactions. Specifically, the authors obtained enhanced reaction rates in the Friedel–Crafts alkylation of indoles with enals under fully aqueous conditions (Figure 11). The peptides were mounted over PEG-PS resin. The introduction of two L-configured Trp residues in the β-turn region of the peptides had a large impact on catalyst performance (85% yield and 88% e.e. for (*S*)) [71]. The reaction outcomes were improved (92% conversion and 91% e.e.) by introducing two units of the Leu-Leu-Aib triad within the polyLeu sequence, and this result was attributed to the stabilization of a 3^10^-helix rather than the α-helix [72].

The peptide Pro-D-Pro-Aib-Trp-Trp-(Leu-Leu-Aib)_2_ was also supported onto TentaGel S-NH_2_ resin to perform the asymmetric conjugate addition of an arylthiol to α,β,γ,δ-unsaturated aldehydes, in the presence of a catalytic amount of acid (e.g., TFA). The reaction gave the kinetically favored 1,4-adduct, but these products were eventually converted to thermodynamically stable 1,6- and 1,4-diadducts through retro-addition/addition reactions. This led to high conversion (up to 99%), albeit the diadducts showed a certain tendency to racemization(up to 79%, e.e.) [73].

**Figure 12:**
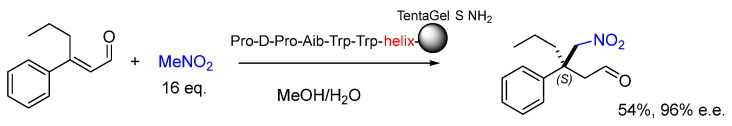
Supported β-turn-helical peptide catalyst for conjugate addition of nitromethane.

On the other hand, the same peptide Pro-D-Pro-Aib-Trp-Trp-(Leu-Leu-Aib)_2_, attached to a helical polypeptide and supported on TentaGel S-NH_2_, was utilized also in the conjugate addition of nitromethane to β,β-disubstituted α,β-unsaturated aldehydes [74]. The reaction conditions had to be carefully re-examined, but eventually, the peptide effectively promoted the reaction in aqueous media with high enantioselectivity (e.g., 54% yield and 96% e.e. for (*S*), for the example reported in Figure 12 in MeOH/H_2_O).

**Figure 13:**
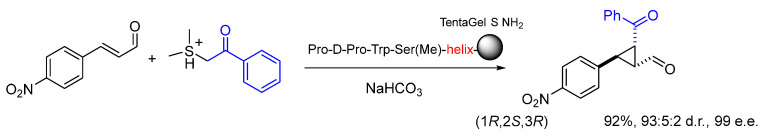
Supported β-turn-helical peptide catalyst for cyclopropanation of enals.

The same type of catalyst was utilized for the asymmetric cyclopropanation of α,β-unsaturated aldehydes with sulfur ylides [75]. The β-turn motifs of sequence Pro-D-Pro-Aa1-Aa2-, attached to a helical (Leu-Leu-Aib)_2_ segment, were supported on TentaGel S-NH_2_. The authors confirmed that the β-turn was crucial for controlling enantioselectivity, while the hydrophobic helical portion stabilized the peptide in the aqueous reaction medium. Incorporation of Trp as Aa1 and Ser(Me) as Aa2 gave the best overall results (92% conversion, 93:5:2 d.r. and 99% e.e. for (1*R*,2*S*,3*R*)), with both electron-rich and electron-poor α,β-unsaturated aryl aldehydes (Figure 13). The reusability of the resin-supported catalyst was also examined, and no significant decrease in catalytic activity was observed even after the fifth cycle.

**Figure 14:**
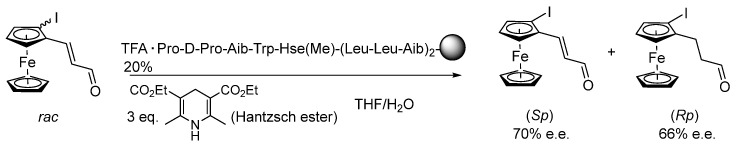
Kinetic resolution of planar-chiral enal-ferrocenes by reduction of the unsaturated bond.

Kudo and co. further applied as catalysts Pro-D-Pro-Aib-Trp peptides connected to the helix-forming (Leu-Leu-Aib)_2_ unit, supported on TentaGel S-NH_2_, in the kinetic resolution of chiral, planar, enal-containing ferrocene complexes, via asymmetric reduction of racemates with Hantzsch ester. Unlike other β-turn-helix-peptide catalysts, which relied on an α-helical polyLeu chain, the (Leu-Leu-Aib)_2_ motif was seen to form a 3^10^-helix. The presence of methyl-protected homoserine (HseMe) at position i + 4 had a positive effect on selectivity (up to 70% e.e. for stereoisomer (*Sp*), up to 66% e.e. for stereoisomer (*Rp*)) (Figure 14). Much better results were obtained when the kinetic resolution was performed through the Michael addition of nitromethane (up to 84% e.e. for stereoisomer (*Sp*), up to 92% e.e. for stereoisomer (*S*,*Rp*), with 98:2 d.r.) [76], albeit reaction yields in some cases were moderate (up to 55%).

The use of solid-supported Pro-Aib-based peptides was also exploited for the α-functionalization of aldehydes. The α-amination reactions of aldehydes were performed with diisopropyl azodicarboxylate. The β-turn forming motif D-Pro-Aib-Phe provided the highest enantioselectivity (with benzaldehyde, 99% yield, 98% e.e.). The catalyst was supported onto PEG-PS resin so it could be easily recovered by filtration and reused ten times [77]. Interestingly, the absolute sense of asymmetric induction was mainly controlled by the configuration of the N-terminal Pro residue.

**Figure 15:**
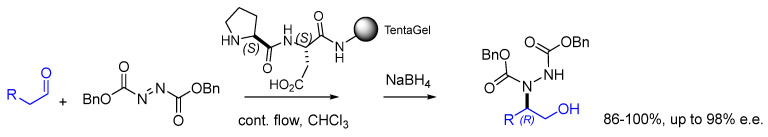
α-Amination of aldehydes catalyzed by a supported Pro-peptide.

Fülöp and co. insisted in this direction and developed a continuous-flow type process to produce amino alcohols from aldehydes and azodicarboxylate in a high-throughput manner on a gram-scale (up to 98% e.e. for (*R*) and 86-100% yield). Initially, the authors utilized PS resin with a 4-MBHA linker (PS-MBHA). The catalyst carrier was changed from PS-MBHA to TentaGel, which appeared to be more robust and stable to pressure under the experimental flow chemistry conditions (Figure 15). The immobilized peptide column was found to be durable, with no signs of decrease in er or efficiency even after 20 consecutive hours of use [78].

**Figure 16:**
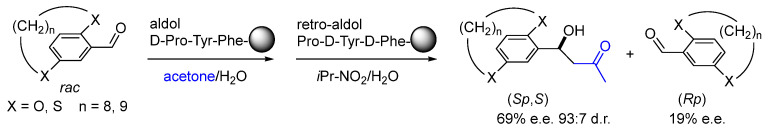
Kinetic resolution of ansa-cyclophanes by sequential aldol/retro-aldol reaction, both catalyzed by supported peptides.

The kinetic resolution of racemic ansa-cyclophanes (13-formyl-1,11-dioxa[11]paracyclophane, i.e., cyclophanes with an aliphatic bridge) was accomplished via a sequential aldol/retro-aldol process. To this purpose, Kudo and co. used the peptide D-Pro-Tyr-Phe and its enantiomer Pro-D-Tyr-D-Phe, both supported onto PEG–PS resin. In the reaction of formyl-cyclophane with acetone, the (*Sp*) isomer resulted in good stereoselectivity for the (*Sp*,*S*) aldol (69% e.e., 93:7 d.r.), with moderate conversion (up to 26%), and in scarce enantiomeric excess for the unreacted (*Rp*) formyl-cyclophane (19% e.e.) (Figure 16). Also, the selectivity was found to slightly diminish after just two reuses [79].

For the conjugate addition of nitromethane to enones, a resin-supported peptide containing an N-terminal Trp-Trp diad instead of a Pro-peptide and (Leu-Leu-Aib)_3_ as 3^10^-helix-promoting linker region, was identified as the lead catalyst, as it afforded the product in 93% yield and 91% e.e. [80].

## 5. On-Bead Immobilization and Screening of Peptide Catalysts

The OBOC method has often proved to be very productive for screening large libraries of potential peptide catalysts, allowing for the identification of potential hits to be utilized both supported and unsupported. The libraries were generated and assayed by combinatorial, split-and-pool method. After synthesizing large arrays of on-bead peptide libraries, each bead can be manually sorted into individual reaction vessels and screened for activity using GC or HPLC to assess conversion and stereoselectivity. The on-bead peptides with the best efficiencies can then be analyzed, for instance, partial Edman degradation-mass spectrometry. Possibly, upon identification of hit sequences, the peptides can also be evaluated in homogeneous catalysis.

The first example discussed here is the direct site-selective epoxidation of the polyene substrate farnesol. Aspartic acid-containing peptides were screened by OBOC procedure. To this purpose, the sequences were supported either on a PEG-PS-NH_2_ resin or a PS resin equipped with a linker constructed from three aminohexanoic acid monomers.

**Figure 17:**
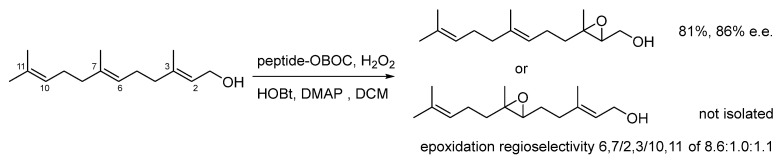
Identification of peptide catalysts for regioselective epoxidation.

Interestingly, diverse peptides consented to preferentially obtain alternative regioselectivities. The best hits were resynthesized and tested also in solution (Figure 17). The peptide Boc-Asp-Pro-Asn(Trt)-D-Phe-Asn-Pro-Asn(Trt)-OMe consented to increased regioselectivity to >100:1:1 in favor of 2,3-epoxydation (86% e.e. for (*S*,*S*)) [81]. In contrast, Boc-Asp-D-Pro-Thr(Bn)-Asn(Trt)-Tyr(tBu)-Gly-OMe favored the reaction in 6,7, giving the epoxide with a 6,7/2,3/10,11 ratio of 8.6:1.0:1.1 [82].

The Ball group incorporated rhodium into peptide scaffolds to obtain hybrid monomeric or dimeric catalysts. To accelerate the discovery of selective ligand sequences for the asymmetric cyclopropanation of styrenes with α-diazophenylacetate, a library of peptides was screened by OBOC method, using PEG-PS resin Novasyn TGR. However, the on-bead screening gave contrasting and unreliable results, albeit eventually, some highly efficient catalysts for in-solution chemistry were identified [83,84].

**Figure 18:**
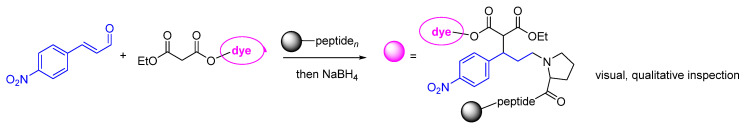
Reaction between dye-marked malonate and an α,β-unsaturated aldehyde in the presence of resin-supported peptide catalysts. After reaction, colored resin beads were considered indicative of peptide catalysts, which effectively promoted the reaction.

As an improvement to the OBOC procedure, Kudo and co. entrapped on beads, the iminium ion formed as an intermediate of Pro-peptide-catalyzed conjugate additions. Using a dye-modified malonate as a nucleophile, 4-nitrophenylacrylaldehyde as an electrophile, and a peptide catalyst supported on TentaGel S-NH_2_, the intermediate Pro-iminium ion was reduced to give a resin-bound conjugate addition product (Figure 18) [85,86]. Hence, this strategy was used to covalently append a dye molecule to a catalytically active peptide. Upon washing excess dye, beads that remained colored were considered suggestive of the presence on these beads of peptides with high reactivity.

## 6. Immobilization on Inorganic Supports

The availability of ordered mesoporous silica materials disclosed a wide range of opportunities for catalyst immobilization [87,88]. In general, the pore size of these materials can be tuned in the range of 2-6 nm. Immobilization can be obtained by covalent binding, electrostatic interaction, adsorption, and encapsulation. For easy covalent binding, ordered mesoporous silica (OMS) is often functionalized with silanol reagents such as 3-aminopropyltriethoxysilane (APTES) or 3-isocyanatopropyltriethoxysilane (ICPTES), which consents to form highly stable amide or urea bonds, respectively.

**Figure 19:**
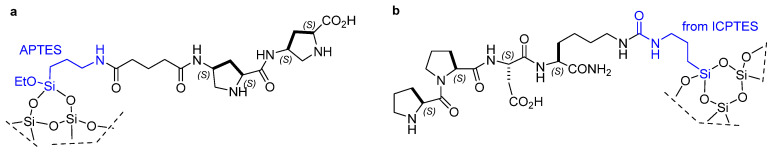
Peptide catalysts supported on silica for aldol reactions, (**a**) anchored via APTES, (**b**) via ICPTES.

Wang and co. functionalized silica with APTES for anchorage on it, a noncanonical diproline catalyst (Figure 19a), which promoted the aldol reaction between acetone and benzaldehyde with high yield (82%) and enantioselectivity (96% e.e.) [89]. With cyclohexanone as the substrate, the catalyst gave the aldol product in quantitative yield (98%), but modest diastereoselectivity (anti/syn 60:40), albeit enantioselectivity in the major diastereomer was remarkable (90% e.e.). It was demonstrated that this catalyst could be recycled up to five times while preserving the observed yield and enantioselectivity.

Utilizing ICPTES chemistry for the functionalization of OMSs, Mehdi, Subra, and co. developed a method for synthesizing hybrid peptide-OMS materials [90]. A Wennemers’ Pro-Pro-Asp peptide catalyst was installed by the C-terminus onto the ICPTES-silica group via urea linker (Figure 19b). This silica-peptide material proved to be a highly selective catalyst in aldol reactions, providing the product with excellent enantioselectivity (>95% e.e.), albeit with moderate yield (59%) as compared to the corresponding homogeneous system. The hybrid peptide-OMS was recovered from the reaction mixture by a simple filtration step. However, the reuse of the recycled OMS in an aldol reaction was not demonstrated.

**Figure 20:**
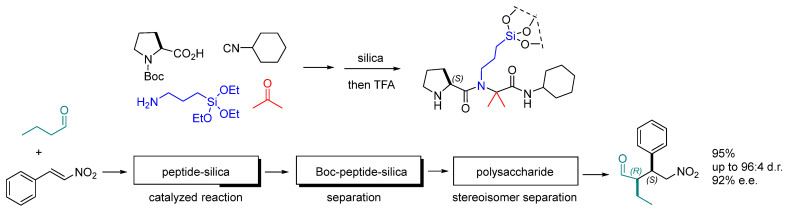
Synthesis of silica-grafted peptide catalyst scaffolds by Ugi reaction and application to subsequent conjugate addition, product isolation, and stereoisomer separation.

Scatena et al. prepared silica-grafted peptide catalyst scaffolds by one-pot multicomponent reaction between proline, acetone, APTES, and cyclohexyl isocyanide, thus giving the simultaneous incorporation of the catalytic and the triethoxysilane moieties. These catalysts were utilized in the asymmetric conjugate addition of aldehyde to nitroolefin in flow chemistry setups (Figure 20). The reactions proceeded smoothly (up to 95% yield, and up to 96:4 d.r., 92% e.e. for (2*R*,3*S*)). A microreactor with high catalytic efficacy and reproducibility was obtained by grafting the silylated peptides onto HPLC-grade silica, and the resulting silica peptides were packed into a column. A 3D continuous-flow system that included online monitoring of the reaction outcome was set up. For that, the microreactor was coupled to a chromatographic column for the separation of the remaining substrates from the Michael adduct in the second dimension. Interestingly, this separation column was filled with a Boc-protected version of the catalytic peptide as a steady phase. The first two columns were followed by a chiral polysaccharide column for the analysis of conversion and stereoselectivity [91].

**Figure 21:**
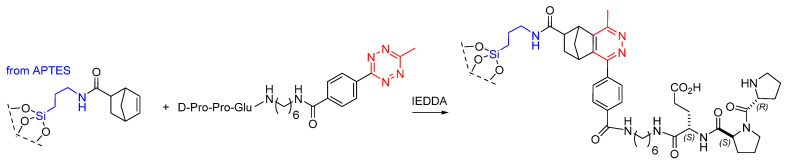
IEDDA ligation between norbornene-silica and peptide-tetrazine.

Very recently, Brand et al. immobilized peptides on different porous silica supports, including commercial mesoporous silica particles with defined pore sizes or monolithic silica possessing mesopores and macropores. The silica supports were functionalized with APTES, then with a norbornene moiety, while the peptide, i.e., D-Pro-Pro-Glu, was modified with a tetrazine moiety, enabling the immobilization onto silica-norbornene via inverse electron demand Diels-Alder (IEDDA) reaction (Figure 21), resulting in catalyst loadings up to 0.2 mmol/g. The model reaction of butanal and nitrostyrene proceeded with excellent yield and selectivity, giving the corresponding nitroaldehyde (97% conversion, syn/anti ratio of 97:3, 96% e.e.) [92].

## 7. Porous Organic Frameworks (POFs)

In the last few years, microporous polymer networks (MPNs), covalent organic frameworks (COFs), and other porous organic frameworks (POFs) aroused some attention as potential supports to be utilized in heterogeneous organocatalysis (see the Introduction). However, very often, these fully organic 3D structures lack functional groups to be utilized as linkers to the organocatalyst. Hence, recent research in the field focused on specific chemoselective strategies for material functionalization.

Lin et al. prepared porous organic frameworks (POFs) carrying Pro as organocatalyst by Ni(0)-catalyzed Yamamoto type Ullmann cross-coupling reaction between tetrakis(4-bromophenyl)methane and Boc-Pro-2,5-dibromophenylsulfonylamide. These Pro-POFs displayed higher catalytic activity and superior enantioselectivity homogeneous proline in the direct asymmetric aldol reactions between nitrobenzaldehyde and acetone (85%, 77.8 e.e.) and could be recycled for at least five times [93].

**Figure 22:**
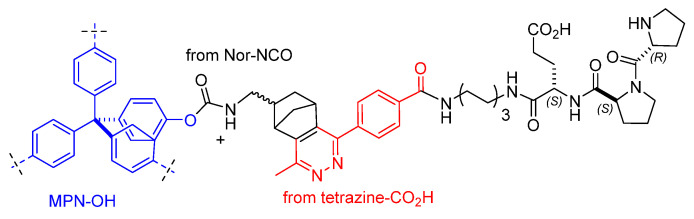
MPN-peptide catalyst obtained by IEDDA ligation between MPN-norbornene and peptide-tetrazine.

Busche et al. synthesized an aromatic microporous polymer network (MPN) with one distinct hydroxy functionality per repeating unit. Specifically, protected 4-methoxyphenyl-tris(4-bromophenyl)-methane monomer was polymerized by Yamamoto coupling reaction, affording the methoxy-protected polymer MPN-OMe, which was subsequently deprotected to MPN-OH with BBr_3_/H_2_O. The hydroxy groups of MPN-OH were reacted with norbornene isocyanate (Nor-NCO) and dibutyltin dilaurate as catalysts to form MPN-norbornene. On the other side, 1,2,4,5-tetrazine-CO_2_H was activated with N-hydroxysuccinimide and coupled with D-Pro-Pro-Glu-hexamethylenediamine to give D-Pro-Pro-Glu-hexamethylenediamine-tetrazine. Hence, the norbornene handles were reacted with the tetrazine to furnish the MPN-peptide catalyst (Figure 22) [94]. The heterogeneous peptide catalyst was tested in the enamine-catalyzed reaction of butanal with nitrostyrene. After 5 recycles, the conversion decreased (from 95% to 73%), while the syn/anti ratio and enantioselectivity remained constant (97:3 and 90% e.e, respectively).

## 8. Metal–Organic Frameworks (MOFs)

Metal–organic frameworks (MOFs) are crystalline, porous organic/inorganic hybrids constructed from relatively simple organic building blocks, which act as connectors between metals (see the Introduction). In particular, MOFs constituted by a metal–peptide framework attracted much interest as potential artificial metalloenzymes [95]. These peptide-MOFs can be obtained either by self-assembly using amino acid or by postsynthetic functionalization.

**Figure 23:**
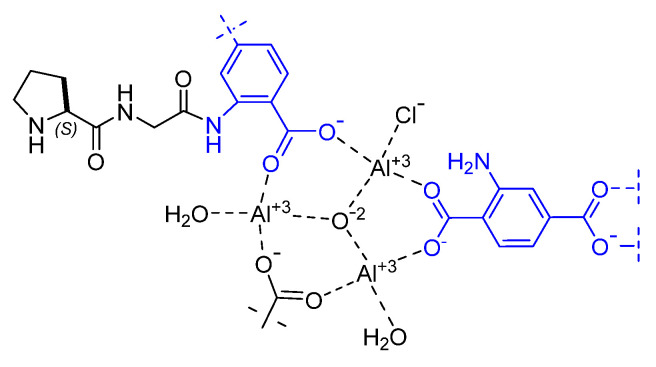
Al-MIL-101 MOFs functionalized with oligopeptide as catalyst for aldol reaction.

Aiming at reproducing enzyme-like complexity, Canivet and co. proposed the first example of MOFs post-functionalization with amino acids or oligopeptides [96]. The MOF platforms Al-MIL-101-NH_2_, In-MIL-68-NH_2_, and Zr-UiO-66-NH_2_ equipped with a 2-aminoterephthalate linker were functionalized with the C-terminus of the peptides using microwave-assisted amide bond formation, resulting in peptides anchored inside MOF cavities (Figure 23). As a proof-of-concept, the peptide-MOF Al-MIL-101-NH-Gly-Pro was employed as a catalyst for the aldol reaction between 4-nitrobenzaldehyde and acetone. Despite the low enantioselectivity (17% e.e.), the product was obtained in almost quantitative yield.

Fracaroli et al. expanded the range of post-synthetic covalent modification allowed within the pore of a MOF without loss of structural integrity and structural organization [97]. The authors utilized MOFs composed of magnesium oxide rods joined by terphenylene organic struts and eventually functionalized the material with Cys-His-Asp peptide for selective cleavage of Ala-Ser peptide bond in the pentapeptide Ala-Tyr-Ala-Ser-Ala-CONH_2_. After 24 h, the expected cleavage product Ala-Tyr-Ala was observed, but only in traces.

More recently, Martí-Gastaldo and co. prepared a MOF structure composed of a Cu^2+^-Gly-His-Lys peptide framework. The structure was stabilized by coordination between copper, the imidazole ring of His, the amine of Gly, and the first amide bond [98]. The free amino group of Lys was able to activate guest benzaldehyde and nitroalkane molecules during 3 cycles of Henry reaction, affording β-nitrostyrene (95% yield) under solvent-free conditions.

## 9. Adsorption or Inclusion of Peptides in Inorganic or Hybrid Materials

In alternative to covalent binding, the catalysts can be loaded via surface adsorption, mainly mediated by electrostatic interaction or weak van der Waals interactions. Alternatively, the catalysts can be captured through a reconstruction method—where the material is deconstructed, typically involving the collapse of its microstructure (e.g., by calcination) or partial dissolution/disassembly—and then reconstructed in the presence of the peptide catalyst, allowing the catalysts to become entrapped as the original structure is rehydrated and restored. Leaching of catalysts remains one of the critical challenges in the design of hybrid catalytic systems where organocatalysts are adsorbed or embedded within porous frameworks and not covalently bound.

**Figure 24:**
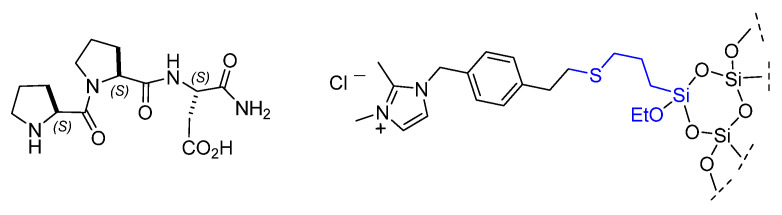
Absorption of Wennemers’ aldol peptide catalyst in silica-imidazolium.

Gruttadauria and co. utilized Wennemers’ previously reported aldol catalyst Pro-Pro-Asp in developing recyclable materials. The peptide was adsorbed onto the surface of silica gel functionalized with 1,2-dimethyl-imidazolium salts (Figure 24). In the first use of the supported catalyst for the aldol reaction of 4-nitrobenzaldehyde and acetone, the β-hydroxyketone product was obtained in nearly quantitative yield (99%) and good enantioselectivity (86% e.e.), results comparable to those obtained under homogeneous conditions. However, both yield and enantioselectivity diminished over the course of a few subsequent runs with the recycled catalysts (38% yield, 83% e.e.) [99].

Further efforts to render peptide catalysts more easily separable from reaction mixtures were envisaged by Segarra and co., who reported the immobilization of polyLeu peptides on hydrotalcite. Hydrotalcite is a Mg/Al hydroxycarbonate with the general formula Mg_6_Al_2_CO_3_(OH)_16_-tetrahydrate, presenting a layered crystal structure. Synthetic polypeptides were immobilized in less than 30 min by ultra-sonication. The immobilization of poly amino acids onto the cationic-charged layered materials was favored by the presence of the carboxylate groups. The catalysts were tested in the epoxidation of chalcone, which proceeded with good yield and stereoselectivity (best result 99%, 93%, e.e.). The nanohybrid materials did not require any preactivation time and were easily reutilized in subsequent reactions without change in activity [100].

**Figure 25:**
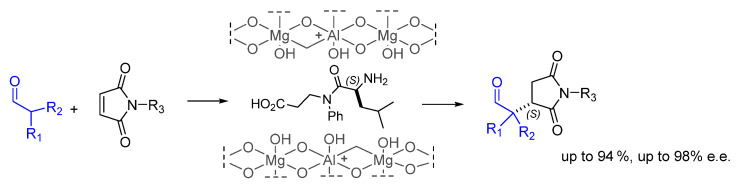
Addition of branched aldehydes to maleimides promoted by dipeptides included into Mg/Al-hydrotalcite layers.

Juaristi and co. developed a method for the intercalation of α-amino acids or hybrid α,β-dipeptides into the interlamellar space of Mg/Al-hydrotalcites (Figure 25). To incorporate the peptides, the crystals were reconstructed by mechanochemical milling, ultrasound activation, and mechanical stirring. This material was designed in the belief that the reactions could provide an augmented catalytic effect in the confined space secluded by the layered material. Remarkably, excellent results (i.e., up to 94 % yield and up to 98% e.e. for (*S*)) were obtained in the addition of branched aldehydes to maleimides even under solvent-free conditions, and the hybrid catalysts could be recycled and reused also in gram-scale experiments [101].

The asymmetric Michael addition of aldehydes to trans-β-nitrostyrene derivatives was conducted by Szőllősi and co. in the presence of amino acids or dipeptides adsorbed on laponite RD. Laponite is a synthetic clay, i.e., a Li/Na/Mg silicate of formula Na_0.7_ Si_8_Mg_5.5_Li_0.3_O_20_-tetrahydrate. The best results were reached with proline-laponite, which gave an increase of stereoselectivity (up to 99%), while dipeptides gave inferior results (for Gly-Pro, 55% yield, 64% e.e.). Characterization of the chiral hybrid material indicated anchoring of the proline to the surface of the laponite particles, with the involvement of both the carboxylic acid and the amino group. As a result, Michael’s addition with Pro adsorbed on laponite provided the opposite enantiomer as compared to proline alone [102].

Very recently, Wennemers’ Pro-Pro-AspNH_2_ peptide was expediently adsorbed on nanocrystalline hydroxyapatite (HAp) without pre-preparation of the solid catalyst. Previously, HAp was proposed as a biocompatible, reusable inorganic base for the synthesis of oligopeptides in a green chemistry perspective [103,104]. HAp belongs to the apatites group with the formula Ca_10_(PO_4_)_6_(OH)_2_ and crystallizes in a hexagonal system. It displays the presence of surface Ca^2+^ and PO_4_^3-^ ions that serve as effective adsorption sites. As a result, HAp has a great affinity for Asp or Glu and for charged peptides.

**Figure 26:**
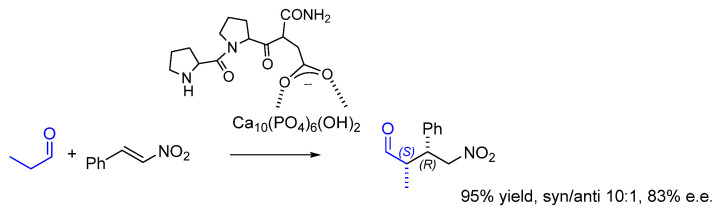
Conjugate addition promoted by Wennemers’ peptide adsorbed on nanocrystalline hydroxyapatite.

The peptide/HAp mixture was utilized for the conjugate addition reactions, with diastereo and enantioselectivity only slightly inferior to the use of the peptide under homogeneous conditions (i.e., 95% yield, syn/anti 10:1, 83% e.e. for (2*S*,3*R*)) (Figure 26) [105].

The hybrid material was readily recovered by centrifugation. Plausibly, the ionic interaction between peptide-carboxylate and calcium ions was fundamental in stabilizing the hybrid material. Indeed, no leakage was observed for the peptide-acid Pro-Pro-AspNH_2_, and the catalyst could be reutilized several times with a moderate loss of activity, while the peptide-amide Pro-Pro-AsnNH_2_ showed significant leakage, preventing its recycle and reuse.

## 10. Conclusions

To sum up, the immobilization of an organocatalyst on a solid support like a polymer or porous silica or its non-covalent absorption consents several clear benefits that improve the overall effectiveness and sustainability of catalytic reactions. This approach allows for straightforward catalyst recovery and reuse, which helps minimize waste and lower operational costs-key advantages for industrial-scale processes. It can also enhance the catalyst’s durability during reactions and reduce the risk of contamination in the final product. Furthermore, immobilized catalysts are well-suited for continuous flow systems, supporting more efficient and environmentally friendly chemical production.

In some experiments, it is apparent that the performance of a peptide catalyst immobilized on a solid support is enhanced due to synergistic interactions between the reagents, the catalyst, and the support material itself. This phenomenon can occur for several reasons. Dual activation: the support material may participate in the catalytic process by interacting with the reagents. For example, it might help orient or activate the reactants via non-covalent interactions, such as hydrogen bonding, electrostatic interactions, or π–π stacking, making them more reactive or better aligned for the peptide catalyst to act upon. Microenvironment effects: immobilization can create a unique local environment around the catalyst. The support may influence parameters like local polarity, pH, or hydrophobicity, which can enhance the peptide’s catalytic activity compared to its behavior in homogeneous conditions. Stabilization of reactive intermediates: the material might help stabilize high-energy intermediates or transition states during the reaction. This stabilization lowers the activation energy and increases the reaction rate. Spatial confinement and pre-organization: in porous or structured supports, reagents and catalysts are brought into closer proximity or confined within a limited space. This confinement can enhance the frequency of productive collisions and improve overall catalytic efficiency. Reduced catalyst deactivation: the support can prevent peptide deactivation by reducing aggregation, protecting sensitive functional groups, or facilitating easier removal of byproducts.

## Data Availability

No new data were created or analyzed in this study.

## Abbreviations

The following abbreviations are used in this manuscript:

e.r.	enantiomeric ratio
e.e.	enantiomeric excess
d.r.	diastereomeric ratio
J-C	Juliá–Colonna
DKP	diketopiperazine
SPPS	solid-phase peptide synthesis
PEG	polyethylene glycol
PS	polystyrene
OMS	ordered mesoporous silica
CuAAC	Cu-catalyzed alkyne-azide cycloaddition
MBHA	methyl benzhydryl amine
MPNs	microporous polymer networks
COFs	covalent organic frameworks
POFs	porous organic frameworks
MOFs	metal–organic frameworks
Aib	2-aminoisobutyric acid
